# Effect of Polystyrene Microplastics on Rice Seed Germination and Antioxidant Enzyme Activity

**DOI:** 10.3390/toxics9080179

**Published:** 2021-07-30

**Authors:** Qiuge Zhang, Mengsai Zhao, Fansong Meng, Yongli Xiao, Wei Dai, Yaning Luan

**Affiliations:** College of Forestry, Beijing Forestry University, Beijing 100083, China; Zhangqiuge@bjfu.edu.cn (Q.Z.); zmengsai96@gmail.com (M.Z.); mfs1997@bjfu.edu.cn (F.M.); Xiaoyongli94@bjfu.edu.cn (Y.X.)

**Keywords:** microplastics, rice seeds, germination experiment, antioxidant enzymes, transcription, expression

## Abstract

The accumulation and distribution of microplastics (MPs) in agricultural soils, including rice fields, is well studied. However, only a few studies have investigated the uptake of MPs by rice plants and the consequential toxic effects of MPs under solid-phase culture conditions. Hence, in this study, we explored the effects of different concentrations of polystyrene MPs (PS-MPs, with a size of 200 nm) on rice seed germination, root growth, antioxidant enzyme activity, and transcriptome. PS-MPs exhibited no significant effect on the germination of rice seeds (*p* > 0.05). However, PS-MPs significantly promoted root length (10 mg L^−1^; *p* < 0.05), and significantly reduced antioxidant enzyme activity (1000 mg L^−1^; *p* < 0.05). Staining with 3,3-diaminobenzidine and nitrotetrazolium blue chloride further revealed significant accumulation of reactive oxygen species in the roots of rice treated with PS-MPs. In addition, transcriptome data analysis revealed that PS-MPs induce the expression of genes related to antioxidant enzyme activity in plant roots. Specifically, genes related to flavonoid and flavonol biosynthesis were upregulated, whereas those involved in linolenic acid and nitrogen metabolism were downregulated. These results enhance our understanding of the responses of agricultural crops to MP toxicity.

## 1. Introduction

Thompson et al. [1] first proposed the concept of “microplastics (MPs)” and discussed their potential effects on marine environments. Currently, most research on MPs is focused on aquatic environments, including oceans, rivers, and shoals [1,2,3], with far less attention paid to terrestrial systems. In fact, Rillig and Matthias [4] proposed that terrestrial ecosystems may also be affected by MPs, with others reporting that MP pollution in terrestrial systems may be 4–23 times higher than that in oceans [5]. MPs are characterized by small particle size, large specific surface area, strong adsorption, wide distribution, stable properties, and unclear toxicological effects [6].

Each year, the application of agricultural sewage sludge causes a large amount of plastic to enter agricultural soil. Indeed, the application of agricultural sewage sludge alone results in a major input of MP particles into agricultural soils, estimated to be between 63,000 and 430,000, and 44,000 and 300,000 tons per year in farmlands in Europe and North America, respectively [7], and between 2800 and 19,000 tons per year in Australian agroecosystems [8]. Existing research reveals the accumulation and characteristics of MPs in rice fields and provides important data that warrants the investigation of the ecological risks and food security within rice-fish culture systems [9]. Moreover, compared with soil media, MPs are more mobile in liquids and are more likely to accumulate in plants by transpiration. Meanwhile, due to their strong adhesive and deformative properties, they readily adhere to, and become absorbed by plants. For instance, Li et al. [10] found that submicrometer- and micrometer-sized polystyrene as well as polymethyl methacrylate particles can enter the plant root epidermis through the gap between primary and secondary roots and, subsequently, reach the central system of wheat roots through apoplasts and Kjeldahl band cracks, thus facilitating the transport of NPs to the stems and leaves, where they accumulate. This distribution of NPs in plant stems and leaves allows for their direct ingestion in food products. That is, humans might be consuming more MPs through plant-based foods (grains, vegetables, fruits, etc.). Additionally, intracellular uptake of nanoplastics (20–40 nm) has been demonstrated in tobacco BY-2 plant cell cultures [11]. Accordingly, Sun et al. [12] investigated the toxicity, absorption, and accumulation of polystyrene nanoplastics (PS-NPs) with different particle sizes (66 nm and 77 nm) in *Arabidopsis thaliana*, and found that these particles could be absorbed by root hairs in the mature zone and internalized near the stele through the apoplast pathway. Moreover, Bosker et al. [13] found that MPs (Fluoro-Max Green Fluorescent Polymer Microspheres) at nominal sizes of 50 nm, 500 nm, and 4800 nm accumulated in the pores of plant seed sacs, thus delaying seed germination and root growth. Still further, Liao et al. [14] showed that 100 nm and 5 µm polystyrene MPs (PS-MPs) could significantly inhibit root and stem elongation in terrestrial higher plants such as wheat (*Triticum aestivum*), and damage the photosynthetic system of leaves and inhibit antioxidant enzyme activities. Meanwhile, Liu et al. [15] found that polyethylene MPs (0.55–0.88, 0.106–0.15, and 0.023–0.038 mm) did not significantly reduce the germination percentage of mung beans, indicating inconsistencies within the current research on the toxic effects of MPs in plants. To date, selected research groups have conducted studies on the impact of MPs on plants, leading to the postulation that MPs can induce various adverse effects on organisms, including disruption of feeding, reproductive reduction, intestinal damage and metabolic disturbances [16,17,18,19,20,21]. Thus, engineered nanoparticles (NPs) may pose threats to human health through the food chain [22,23]. The research on MP toxicity and the underlying mechanisms in plants remains in its infancy, warranting additional studies to enhance our current understanding, particularly in crop plants.

The impact of MPs on rice (*Oryza sativa* L.), a globally important food crop, following their entrance in rice farmlands, is not yet known. In recent years, it has been discovered that MPs of a size of 200 nm can be absorbed into plants [10]. Moreover, a limited number of methods have proven to be effective for the extraction and quantification of MPs; hence, the lack of theoretical support impedes relevant research on the effects of MPs on plant growth under environmental and experimental conditions. Accordingly, the current study sought to explore the effects of 200 nm MPs on rice seed germination, root growth, antioxidant enzyme activity, and transcriptome in indoor hydroponic experiments. The results of the present study provide theoretical data to enhance our understanding of the potential effects of MPs on food crops, particularly rice, facilitating further research on the toxicity-causing mechanisms of PS-MPs in crops.

## 2. Materials and Methods

### 2.1. Preparation and Characterization of Polystyrene Micrometer Plastics

A suspension of 10 mg mL^−1^ original PS-MPs (Da’ e Scientific Co., Ltd., Tianjin, China) was used to prepare working solutions of concentrations 0.1 mg·L^−1^, 10 mg·L^−1^, and 1000 mg·L^−1^. The prepared PS-MP solutions were observed with transmission electron microscopy (TEM) and Fourier-transform infrared spectroscopy (FT-IR). A zeta potential measuring instrument (Malvern Instruments., Ltd., Malvern, UK) was used to detect the zeta potential of the three PS-MP concentrations, whereas chargeability and stability were determined according to the relationships between potential and system stability.

### 2.2. Seed Soaking, Germination, and Cultivation

Clean beakers were autoclaved at 110 °C for 2 h. A total of 600 uniform and full grain rice seeds (*O. sativa* L., ZH11 variety, purchased from the Institute of Genetics, Chinese Academy of Sciences) were sterilized with 3% H_2_O_2_ for 15 min, rinsed with deionized water 5–6 times, and soaked in distilled water or in the three solutions of different NP concentrations for 24 h at 25 °C in the dark. The soaked seeds were then placed into four sterilized 250 mL beakers, 50 seeds were placed in each beaker, and 20 mL of distilled water, as well as three concentrations of 200 nm polystyrene solution, were added to the four beakers. The beakers were placed in an artificial climate box (RXZ-280B, Ningbo Southeast Instrument Co., Ltd., Ningbo, China) for 14 days in the dark at 25 ± 1 °C in an atmosphere with 75% humidity. During the cultivation process, the beakers were weighed every day to account for the lost water volume. To prevent polystyrene plastic particles from gathering, or mildewing of the solution during the cultivation process, the solution was dispersed with an ultrasonic vibrator (JP-010T, Shenzhen Jiemeng Cleaning Equipment Co., Ltd., Shenzhen, China) every day; the solution in each beaker was replaced every 7 days. During this time, the rice seedlings were removed and washed with distilled water. After replacing the seedlings in the beakers, seed germination (roots of the seeds break through the seed coat) was observed and recorded each day.

### 2.3. MP Absorption and Distribution Characteristics

#### 2.3.1. Vacuum Freeze-Drying Method for Scanning Electron Microscopy

After 14 days of cultivation in 1000 mg·L^−1^ PS-MP solution, the rice plant seeds, roots, and buds were collected. The samples were immersed in 2.5% pentanediol, fixed at 4 °C for 3 h, and rinsed with 0.1 mol·L^−1^ phosphate buffer (pH 6.8) three times, for 15 min each. The seedlings were then soaked in 100% pure tert-butanol for 15 min and dried for 5 h at −16 °C in a vacuum freeze dryer (Free Dryer ES-2030, HITACHI, Toxyo, Japan). Conductive tape was used to fix the sample on the scanning electron microscope (SEM; SU8010, HITACHI, Tokyo, Japan) sample stage to ensure the observation surface was flat and oriented upward. The surface of the sample was coated with an ion-sputtering coater for 80 s. The settings for the SEM were as follows: working distance, 8.0; filament current, 20 μA; accelerating voltage, 6 kV; magnification, 200× and 500× [24,25,26].

#### 2.3.2. TEM Embedding

After 14 days of germination, the roots that were 3–4 mm in length and ~1 mm in width were sampled from rice seedlings cultured in 1000 mg·L^−1^ PS-MP solution and soaked in 2.5% glutaraldehyde fixative. The samples were placed in an appropriate mold (e.g., capsule) filled with an embedding agent and cured in an oven at 70 °C for 8 h. The samples were then sliced with an ultra-thin microtome (50–100 nm) and dispersed on a copper mesh. The samples were visualized using a high-contrast transmission electron microscope (JEM 1010, JEOL, Tokyo, Japan) with the following settings: working distance, 8 mm; accelerating voltage, 100 kV; magnification, 1000× [27,28,29].

### 2.4. Determination of Antioxidant Enzyme Activity in Rice Buds

After the 7th day of rice seed culture, the roots were collected, washed with distilled water, and the active oxygen levels and antioxidant enzyme activities were measured. Reactive oxygen species (ROS) were detected using 3,3′-diaminobenzidine (DAB) [30] and nitrotetrazolium blue chloride (NBT) stain [31]. Superoxide dismutase (SOD) activity was measured using the NBT reduction method [29,32,33]. Peroxidase (POD) activity was measured using the guaiacol method [34]. Catalase (CAT) activity was measured using the hydrogen peroxide decomposition method [33,35].

### 2.5. Transcriptomic Determination of the Root System of Rice Seedlings

A 1000 mg·L^−1^ PS-MP solution and distilled water from rice roots (14 days) were collected in a 10 mL RNase free EP tube (Eppendorf Micro Test Tubes) (Shanghai Majorbio Bio-Pharm Technology Co., Ltd., Shanghai, China), frozen with liquid nitrogen, and stored in an ultra-low temperature refrigerator at −80 °C (MDF-U53, Sanyo, Osaka, Japan). The samples were placed in a mortar and finely ground. We then proceeded with a routine eukaryotic mRNA sequencing process by extracting the total RNA and fragment RNA using mRNA as a template to synthesize cDNA and connected adapter and fragment screening and library enrichment to obtain the final library. Finally, an Illumina Novaseq (Shanghai Majorbio Bio-Pharm Technology Co., Ltd., Shanghai, China) machine was used to sequence the transcription products.

In the experiment, high-throughput transcriptome sequencing was performed on rice roots treated with PS-MP solutions and distilled water; 61,586 transcripts were obtained from the sequenced samples. The quality of the generated library and sequencing, at the macro level, was assessed using statistical methods to calculate the base distribution and quality fluctuations of each cycle for all sequencing reads. Additionally, the base quality of each sample, base error rate, and base distribution were analyzed. Six cDNA libraries (CK1, CK2, CK3, PS1, PS2, and PS3) were constructed, and high-throughput sequencing was performed on the Illumina Novaseq (Shanghai Majorbio Bio-Pharm Technology Co., Ltd., Shanghai, China) 6000 sequencing platform to obtain 47769212, 41730978, 41516362, 44105636, 44139948, and 46671662 clean reads. The GC contents of the reads were 54.03%, 54.12%, 54.06%, 53.41%, 53.68%, and 53.6%, respectively, and the Q20 average was over 97%, indicating that they were suitable for subsequent data analysis (Supplementary Table S2).

Pearson’s correlation analysis between the PS treatment group and control group shows a strong correlation between the biological duplicate samples of the PS group and the CK group (>0.9), indicating that the subsequent analyses are credible (Supplementary Figure S1).

Gene ontology (GO) enrichment analysis software Goatools (https://github.com/tanghaibao/GOatools, accessed on 6 January 2020) was used to analyze the genes/transcripts. Specifically, when *p* of the gene set (FDR) < 0.05, the GO function is relevant to the specific gene of interest. Based on the gene expression quantitative results, differentially expressed genes (DEGs) were detected between groups using DESeq2 software. DEGs were assigned if |log2FC| ≥ 1 and *p* < 0.05. The R package was then used to write a script to perform Kyoto Encyclopedia of Genes and Genomes (KEGG) pathway analysis on the genes/transcripts in the gene set. The calculation principle was the same as that described for the GO functional enrichment analysis. When *p* < 0.05, the KEGG pathway was significantly enriched.

### 2.6. Statistical Analysis

The test results were calculated using Microsoft Excel and the data are expressed as the mean ± standard error (SE). The SPSS 25.0 data processing software was used for statistical analysis. The differences in averages between different treatments (*p* < 0.05) was analyzed using the Duncan method, and Origin2019 was used for software mapping. The reference gene source was *Oryza_sativa*, reference genome version was IRGSP-1.0, and the reference genome source was http://plants.ensembl.org/Oryza_sativa/Info/Index, accessed on 6 January 2020. The clean reads for each sample were compared with the designated reference genome, and the comparison rate ranged from 90.62% to 92.75%.

## 3. Results and Discussion

### 3.1. Basic Properties of PS-NPs

TEM was used to characterize the basic properties of PS-MPs (Supplementary Figure S2). The PS-MPs used in the experiments had uniform particle sizes, good dispersibility, and clear spherical shapes. In addition, FT-IR analysis of the PS-MP solutions showed that the peaks appear at 717.450 cm^−1^, 1471.544 cm^−1^, and 2916.087 cm^−1^, which were identified as the infrared absorption spectrum for -CH_2_- in-plane swing, -CH_2_- variable-angle vibration, and -CH_2_-antisymmetric stretching, respectively. The spectra of PS-NPs used in the experiments had high similarity to those of standard PS-MPs (Infrared Spectrum Database of Shanghai Institute of Organic Chemistry), and the two showed similar peak characteristics at the same wavelength (Supplementary Figure S3), indicating that standard PS-MPs were used in these experiments. The particle size of the PS-NPs was 200 nm; the particles were negatively charged, and PS-MP solution stability increased with an increase in solution concentration (Supplementary Table S1).

### 3.2. Effect of PS-NPs on the Germination of Rice Seeds

Germination percentage (GP), germination index (GI), vigor index (VI), germination vigor (GV), and mean germination time (MGT) are key indicators used to measure the germination ability of seeds under different conditions [5,36]. Compared with those of the control, no parameters changed significantly, which is consistent with the findings of previous studies. For example, Bosker et al. [13] found that 50 nm, 500 nm, and 4800 nm MPs (Fluoro-Max Green Fluorescent Polymer Microspheres) had no significant effect on the germination rate after 24 h of seed germination.

**Figure 1:**
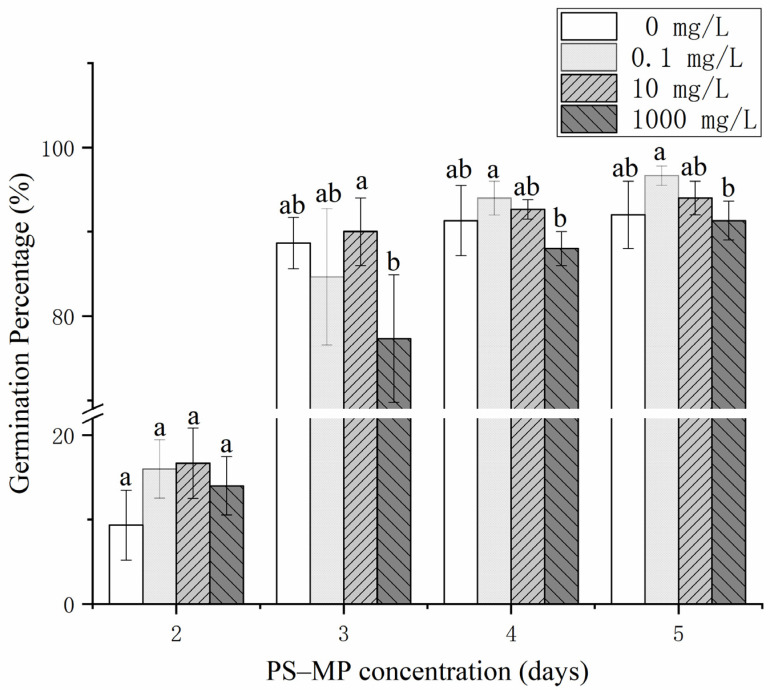
Effects of different polystyrene microplastics (PS-MP) concentrations on rice seed germination. Note: Data are expressed as mean ± standard error (SE). Lowercase letters indicate significant differences between the germination rates of rice seeds treated with different PS-MP concentrations over the same incubation period (*p* < 0.05), ab represents no significant change from a and b respectively. No rice seeds germinated on the first day. The germination rate of the rice seeds in each treatment stabilized on the fifth day. Germination percentage = (number of seeds germinated on the day/number of test seeds) × 100%.

**Figure 2:**
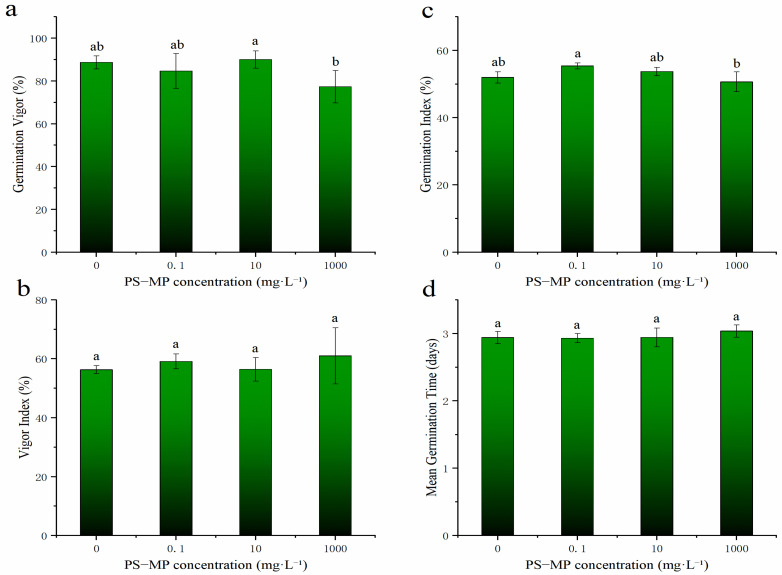
Effects of polystyrene microplastics (PS-MP) concentrations on the germination of rice seeds. (**a**–**d**) are the changes of rice seed germination vigor, vigor index, germination index and average germination time. Note: Data are expressed as the mean ± standard error (SE). Lowercase letters indicate significant differences between the germination rates of rice seeds treated with different PS-MP concentrations (*p* < 0.05), ab represents no significant change from a and b respectively. Germination index (GI) = Σ Gt/Dt, where Gt is the number of germinations in t days; Dt is the corresponding germination days. Vigor index (VI) = germination index × dry quality of seedlings (Supplementary Figure S4); Germination vigor (GV) is the germination rate on the third day; Mean germination time (MGT) = Σ (F × X)/ΣF, where F is the number of new germinations of the seed on day X and X is the number of days of germination.

The GP, GV, and GI of rice seeds exposed to high PS-MP concentrations (1000 mg·L^−1^) were significantly inhibited compared with those at low concentrations, whereas PS-MPs had no significant effects on GI and MGT; the final germination rates of the rice seeds exceeded 91% (Figure 1 and Figure 2 and Supplementary Figure S7).

Early studies on the effects of MPs on plant seed germination reported that a large proportion of MPs enriched in the seed coat may block pores, inhibit water absorption, and retard seed germination [13,37], which are the primary causes for the significant inhibition of the GP of rice seeds under high concentrations of PS-MPs. Overall, compared with the control, the results showed “low promotion and high suppression,” that is, PS-MPs at low concentration promoted the germination of rice seeds, while at high concentration, they inhibited it, although the effect was not significant. This result agrees with those of Foolad et al. [38], who reported that various plants have unique stress tolerance genes or physiological mechanisms that maintain seed germination under certain environmental stresses. Collectively, these results indicate that there is a threshold regarding the effect of PS-MPs on seed germination and provide a reference for the application of nano-fertilizers in agriculture.

### 3.3. Effects of PS-MPs on the Growth of Rice Roots and Buds

**Figure 3:**
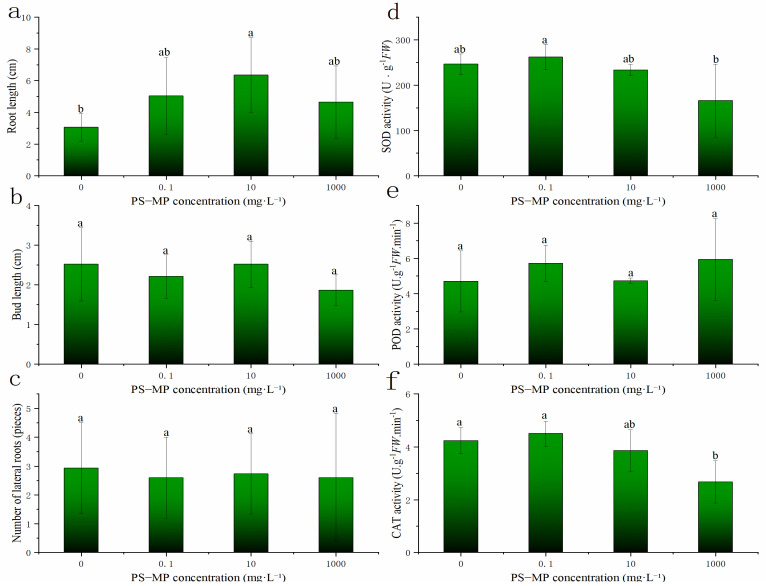
The three panels on the left (**a**–**c**) illustrate the effects of different polystyrene microplastics (PS-MP) concentrations on rice root length, bud length, and lateral root number. The three panels on the right (**d**–**f**) illustrate the effects of MPs on superoxide dismutase (SOD), peroxidase (POD), and catalase (CAT) activity in rice buds. Note: Vernier calipers was used to measure the lengths of rice roots and shoots during rice germination, and the number of lateral roots was counted. Data are expressed as the mean ± standard error (SE). Lowercase letters indicate significant differences between the germination rates of rice seeds treated with different PS-MP concentrations (*p* < 0.05), ab represents no significant change from a and b respectively.

The root lengths of rice treated with different concentrations of PS-MPs were higher than those in the CK and increased significantly under the medium concentration (10 mg·L^−1^). However, PS-MPs had no significant effect on bud length or lateral root number. As can be seen in Figure 1 and Figure 3 (left), high concentrations of PS-MPs (1000 mg·L^−1^) were relatively unsuitable for rice seed germination as well as the growth and development of seedlings compared to low and medium concentrations (Figure 1 and Figure 3a–c). Early research results showed that nano-treatment of wheat with 150 mg/L water-soluble carbon for 10 days could promote root and stem growth [39]. This may be due to the PS-MPs acting as a physical stressor to seeds. PS-MPs are small and accumulate in the epidermis of seeds and roots (Supplementary Figure S3). Early studies reported that plastic particles accumulate particularly in the pores of the seed coat of *L. sativum*. Deposits attached to the surface of the pores slow down water uptake, as observed in soybean (*Glycine max*) seeds [37]. This suggests that clogging of pores with plastic particles might inhibit water uptake, thereby delaying germination. Thus, given that we observed increasingly pronounced effects with the increased size of the plastic, the delay in germination might be caused by physical blocking [13,40].

Root structure also plays a key role in crop productivity and is an important factor in stress resistance [41]. Roots enable plants to perceive changes in external conditions and adapt by altering their root structure. Consequently, crop productivity can be affected by root structure under various environmental stress factors (e.g., the uptake of water and nutrients) [42]. Considering the repressed rice growth and development under PS-MP stress, root growth and development was stimulated to satisfy the nutrient absorption requirements during rice growth. Studies have shown that nanomaterials increase nutrient absorption requirements to promote plant growth and development [43,44,45]. Panova et al. [46] showed that polyhydroxy fullerenes can promote the elongation of barley roots, potentially to eliminate harmful free radicals.

### 3.4. Absorption and Distribution of PS-MPs in Rice

**Figure 4:**
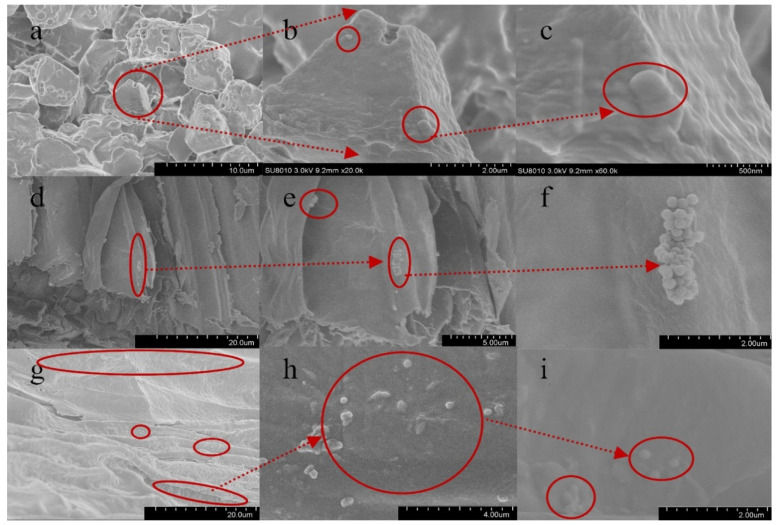
Scanning electron microscopy images of rice seeds, roots, and leaves. Note: the distribution characteristics of PS-MPs in (**a**–**c**) rice seeds; (**d**–**f**) rice roots; and (**g**–**i**) rice buds at a treatment concentration of 1000 mg·L^−1^. (**b**) is a part of (**a**), enlarged; (**c**) is a part of (**b**), enlarged; (**e**) is a part of (**d**), enlarged; (**f**) is a part of (**e**), enlarged; (**h**) is a part of (**g**), enlarged; (**i**) is a part of (**h**), enlarged.

From the relevant rice germination and seedling growth data (Figure 1 and Figure 2), it can be seen that compared with low and medium concentrations, MPs at high concentration are more unsuitable for rice germination and seedling growth. To further determine the influence of PS-MPs on rice seed germination, we first determined the distribution characteristics of PS-MPs in rice. Using SEM (Supplementary Figure S5), we observed that during the germination of rice seeds treated with high concentrations of PS-MPs, there was an obvious enrichment of MP beads on the outer surface of the rice seed epidermis. Most plants have certain stress tolerance mechanisms [38]. Low PS-MP concentrations did not affect seed germination (Figure 1). PS-MPs were distributed in rice seeds, roots, and buds (Figure 4) in the form of granules on the rice seeds and buds (Figure 4a–c,g–i) and clustered along the root surface (Figure 4d–f). These results are consistent with those of Lin et al. [47], who observed that MPs are absorbed from the seeds and roots of plants to the stems and leaves. MPs appear mainly in and around the vascular system of the stem. However, these findings are inconsistent with those reported by Li [10], who found that MP pellets in the plant readily adhere to each other in a chain-like shape, indicating that the MP distribution can take various forms after plant entry. After 14 days of culture, suspected MP particles were observed in root cells (Supplementary Figure S6a,b), indicating that 200 nm PS-MPs could be absorbed by plants at high concentrations (1000 mg·L^−1^) and enter the vascular system through the apoplastic pathway via the cell wall. This is consistent with the results of previous research on the absorption of PS-MPs in *A. thaliana* [12]. We observed that 200 nm PS-MPs could pass through the root epidermis, enter the xylem, be transported into the buds, and even migrate into the cell, which could delay seed germination and stimulate root growth in rice. However, as shown in Figure 4, the absorption of MPs is much lower, which may be related to the secretions of rice roots. Specifically, the root meristem has high cell activity as it regulates cell division and differentiation and is more sensitive to external stimuli. The presence of epidermal cells and border cells also provides the first line of defense for roots against foreign pollutants as the root epidermal cells produce a large number of secretions after external stimulation of the root. This may also explain, in part, why MPs cannot be readily internalized to the root tip [12].

### 3.5. Effects of PS-MPs on the Rice Antioxidant System

#### 3.5.1. Effects of PS-MPs on ROS Levels in Rice Roots

**Figure 5:**
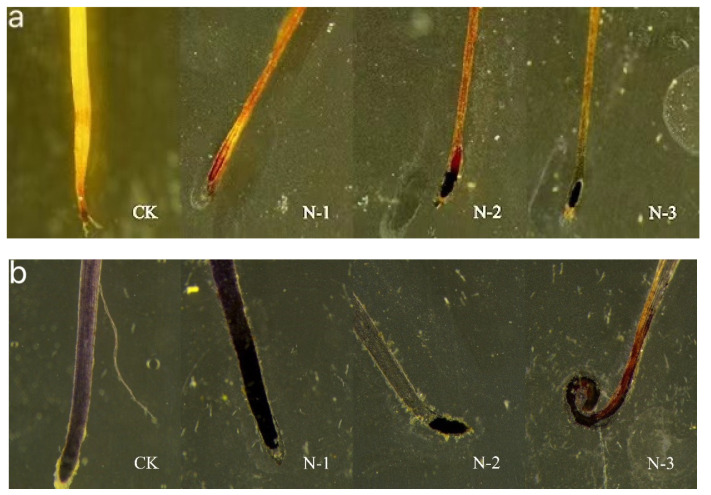
Rice root tips stained with nitrotetrazolium blue chloride (NBT) and 3,3-diaminobenzidine (DAB). Note: (**a**) DAB-stained rice root tip images; H_2_O_2_ reacts with DAB to form reddish-brown spots. (**b**) Root tips stained with NBT; O^2−^ can reduce NBT into a blue precipitate. No microplastics (MPs) added = CK, N-1 = 0.1 mg·L^−1^, N-2 = 10 mg·L^−1^, and N-3 = 1000 mg·L^−1^ NP solution treatments.

ROS are closely related to root growth regulation. Active oxygen is a toxic substance produced during aerobic metabolism in plants [48] and has toxic effects on many physiological activities. Under normal growth conditions, ROS content in plants is relatively stable. However, stress can disrupt this dynamic balance, causing plants to accumulate high amounts of the superoxide anion free radicals, O_2_^−^ and H_2_O_2_, which activate stress responses and defense pathways in plants [49], and enhance plant tolerance to environmental stress. O^2−^ and H_2_O_2_ are key ROSs, the contents of which can indicate the levels of ROS stress in plants. To evaluate the changes in ROS production in response to PS-MPs, we treated rice roots with NBT and DAB and used a stereo microscope to visualize ROS production. Compared with those of CK, the root tips of plants treated with PS-MPs were substantially colored (Figure 5a,b), indicating H_2_O_2_ and O^2−^ accumulation in the roots. This is consistent with the findings of Sun et al. [12]. We also found that the O_2_ and H_2_O_2_ in plants were significantly accumulated. As the PS-MP concentration increased, the reddish-brown product of H_2_O_2_ and DAB became darker, while the blue precipitate formed by O^2−^ and NBT became darker and the root tip curled, indicating that the PS-MP-mediated ROS accumulation in the root tip caused altered root tip morphology.

#### 3.5.2. Effect of PS-MPs on the Antioxidant Enzyme Activity in Rice Buds

Compared with the control, the activities of SOD and CAT showed the phenomenon of “low promotion and high suppression” at different concentrations of PS-MPs, with the activity of CAT being significantly inhibited at high concentrations of PS-MPs. However, the activity of POD at different concentrations of PS-MPs was not significantly affected (Figure 3d–f). Plants can alleviate the damage caused by ROS by regulating the activity of antioxidant enzymes [50]. SOD is a metal-containing enzyme that is ubiquitous in aerobic organisms and is one of the main components of plant antioxidant enzyme systems because it can dismutate excessive and harmful oxygen free radicals in plants. It converts harmful oxygen free radicals into H_2_O_2_, which is then converted into harmless molecular oxygen and water by CAT, thus protecting the cells from oxidative stress [51]. Moreover, CAT and POD are implicated in the removal of H_2_O_2_. Numerous studies have shown that SOD activity is associated with plant tolerance to environmental stresses [52,53,54]. Therefore, the decrease in SOD activity after PS-MP treatment may be one of the main reasons for the accumulation of ROS in rice buds. High POD activity can reflect improved growth and development characteristics of plants, as well as the metabolic state and adaptability to the external environment. PS-MPs had no significant effect on POD activity, which may be the reason for nonsignificant changes in shoot length, lateral root number, and in rice plants treated with PS-MPs. This indicated that rice growth and development, metabolism, and adaptability to the external environment were maintained effectively. CAT catalyzes the decomposition of hydrogen peroxide into oxygen and water, which is present in the peroxisome of the cell and mainly targets H_2_O_2_ produced in the process of photorespiration and fatty acid β oxidation [55]. Compared with that of CK, CAT activity at a high PS-MP concentration (1000 mg·L^−1^) was significantly reduced (Figure 3, right). Under low (0.1 mg·L^−1^) and medium (10 mg·L^−1^) concentrations, CAT activity did not reduce significantly due to the resistance of rice; however, high PS-NP concentrations could overcome rice tolerance, inhibit the synthesis of certain protein molecules in rice, and lead to reduced enzyme activity [56,57].

### 3.6. Transcriptomic Analysis of PS-MPs in Rice Seedling Roots

**Figure 6:**
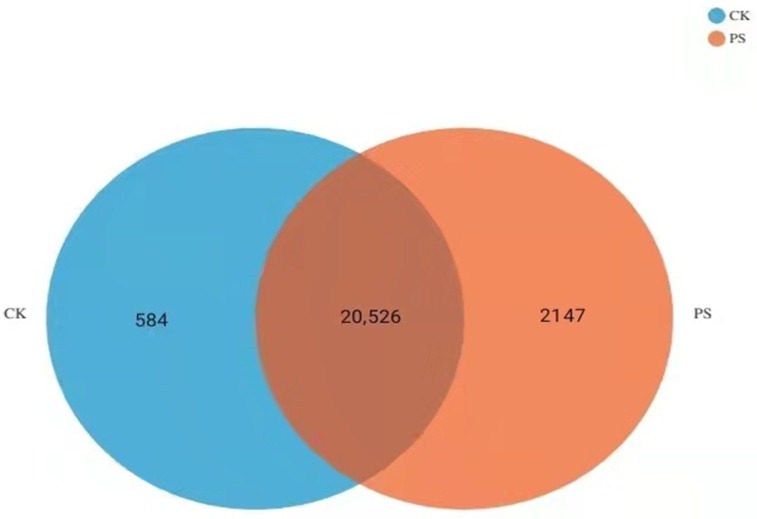
Venn diagram of the specific genes in roots after 14 days of exposure to polystyrene microplastics (PS-MPs).

**Figure 7:**
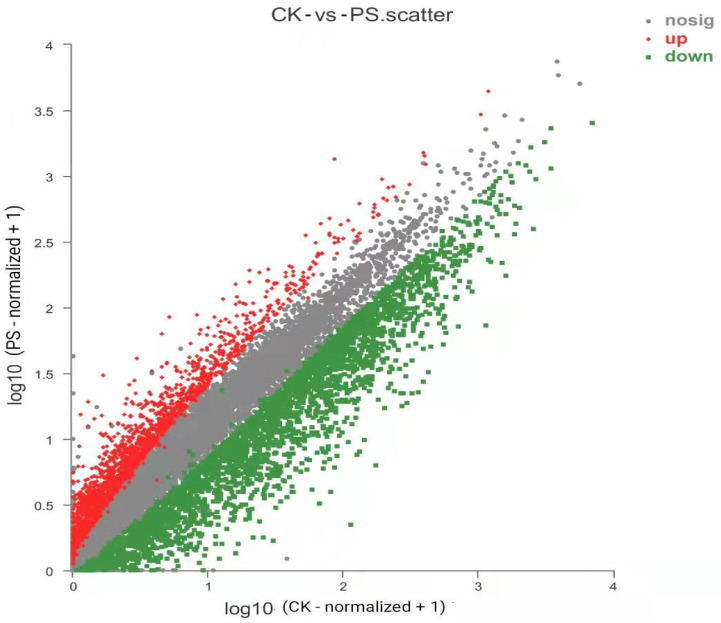
Scatterplot of the distribution of differentially expressed genes (DEGs) in roots after 14 days of exposure to polystyrene microplastics (PS-MPs). Note: upregulated: 1737 (5.36%); nosig: 27,673; downregulated: 3014 (9.3%).

We performed high-throughput transcriptome sequencing on rice roots treated with nanoplastic solutions and obtained 61,586 transcripts from the sequenced samples. The Unigene sequence obtained by sequencing was compared with various gene function databases (NR and SwissProt, pfam protein domain prediction, KEGG metabolic pathway enrichment annotation, and GO metabolic pathway enrichment annotation). Differential analysis of gene expression in rice seedling roots treated with PS-MPs and statistical analysis revealed that there were 584 DEGs in the CK group and 2147 specifically expressed genes in the experimental group treated with 1000 mg·L^−1^ PS-MP solution (Figure 6). Through differential gene screening of the experimental group and CK group, 32,424 genes were found to be differentially expressed, of which 1737 were upregulated, accounting for 5.36% of the total number of DEGs, and 3014 were downregulated, accounting for 9.30% of the total number of genes (Figure 7).

**Figure 8:**
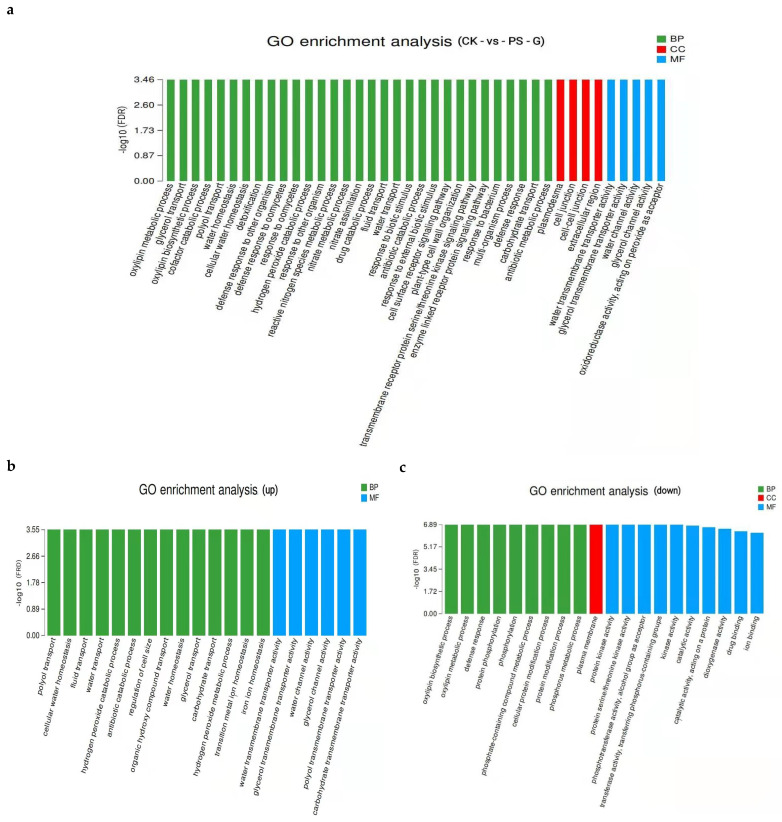
Gene ontology (GO) enrichment classification of differentially expressed genes (DEGs) in rice roots. (**a**) is the enrichment of differential genes, (**b**) is the enrichment of up-regulated genes, and (**c**) is the enrichment of down-regulated genes. Note: The abscissa represents the GO terms, and the ordinate represents the significance level of enrichment, which corresponds to the height of the column. The larger the −log_10_ (FDR) value, the more significant the enrichment of the GO term.

GO functional classification revealed that the 4751 unigenes were divided into 49 functional sub-clusters, out of which 1958 unigenes were classified into the annotation category associated with metabolic processes. Thus, we were able to identify additional genes involved in the synthesis of metabolites in response to MP treatment. We then analyzed the GO enrichment of different genes in rice root systems grown in PS-MP-containing conditions and identified a total of 554 DEGs. The highest number of genes were enriched in the biological process (BP) and the lowest number of genes were enriched in the cell component (CC) (Figure 8a). Among the upregulated genes, there were several gene enrichment clusters, including those enriched in the polyol transport process, cell water homeostasis, liquid transport, hydrogen peroxide catabolism process, cell size regulation, organic hydroxyl compound transport, glycerol transport, and carbon water, which belong to the BP category. Genes were also enriched in compound transport, the hydrogen peroxide metabolism process, transition metal ion homeostasis, transmembrane transport activity of water, glycerol transmembrane transport protein activity, water channel activity, glycerol channel activity, polyol transmembrane transport activity, and carbohydrate transmembrane transporter activity, which belong to molecular functions. The GO terms formed a significant functionally enriched cluster, indicating that the expression of genes involved in these biological processes were actively expressed after treatment of rice with PS-MPs (Figure 8b).

Among the downregulated genes, the main enriched functional clusters were oxylipin biosynthesis, oxylipin metabolism process, defense response, protein phosphorylation, phosphorus-containing compound metabolism process, cell protein modification process, phosphorus metabolism process, category of biological processes, cell membrane and molecular functions of protein kinase activity, serine/threonine kinase activity, phosphatase activity, alcohols as receptors, and enzyme activity for transferring phosphorus-containing groups, indicating that the expression of genes involved in these biological processes was inhibited by PS-MP treatment (Figure 8c).

Collectively, these results suggest that genes involved in the stimulation of antioxidant enzyme activity were downregulated by MPs, and genes related to plant disease resistance were upregulated, which accounted for the marked accumulation of ROS in rice exposed to MPs (Figure 5). However, significant effects on the growth of roots and shoots were not observed (Figure 3).

**Figure 9:**
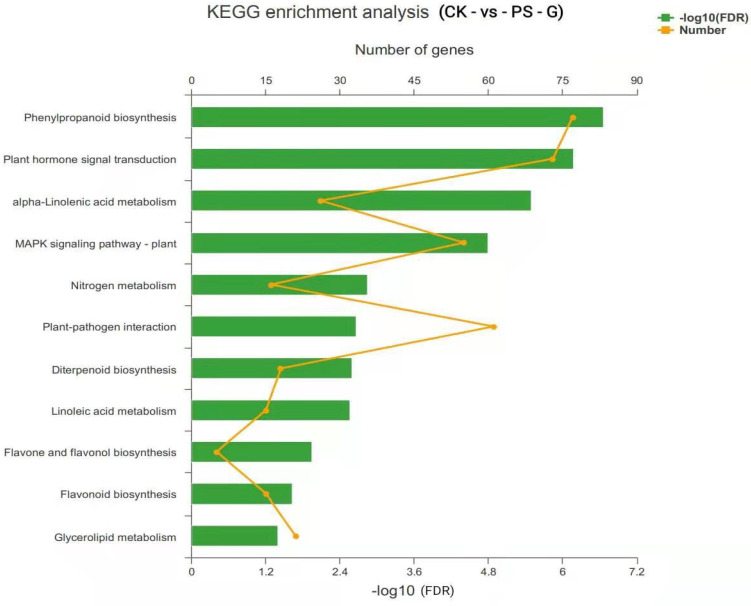
Differentially expressed genes involved in Kyoto Encyclopedia of Genes and Genomes (KEGG) biochemical pathways. Note: The ordinate represents the KEGG pathways, and the upper abscissa represents the number of genes/transcripts of the pathway in the comparison that corresponds to the points on the broken line. The lower abscissa represents the significance level of enrichment, which corresponds to the height of the column, where the greater the −log_10_ (FDR) value, the more significantly enriched the KEGG pathway is.

KEGG metabolic pathway classification identified 332 unigenes participating in 24 major biochemical metabolic pathways, including the biosynthesis of other metabolites, signal transduction, and metabolism of terpenoids and polyketides. Among them, 212 genes were identified as related to metabolic functions, and 33 were identified as related to environmental processing functions (Figure 9). These biochemical metabolic pathways provide insights into the biological functions of rice metabolism and adaptability to the environment under MP treatment.

**Figure 10:**
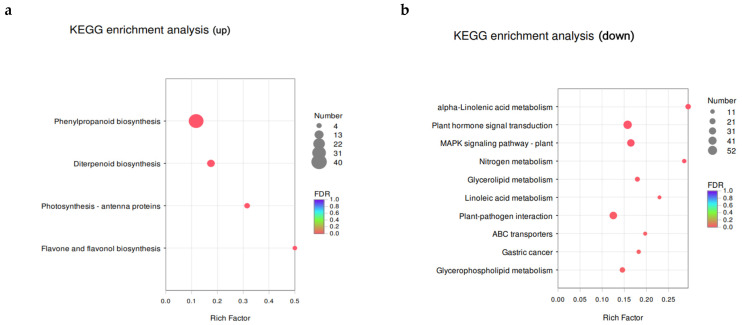
Scatterplot of the Kyoto Encyclopedia of Genes and Genomes (KEGG) pathway enrichment of differentially regulated, differentially expressed genes (DEGs). (**a**) is the analysis of up-regulated genes, and (**b**) is the analysis of down-regulated genes. Note: The vertical axis represents the pathway name and the horizontal axis represents the enrichment factor (the ratio of the number of genes/transcripts enriched in the pathway to the number of annotated genes/transcripts). The larger the enrichment factor, the greater the degree of enrichment. The size of the dot indicates the number of genes in the pathway, and the color of the dot corresponds to different Q-value ranges.

The upregulated genes were primarily enriched in phenylpropane biosynthesis and plant hormone signal transduction metabolic pathways. In addition, the upregulated genes were enriched in the biosynthesis of flavonoids and flavanols as well as the photosynthesis-antenna protein synthesis pathway, which had the largest enrichment factors (0.5 and 0.32, respectively; Figure 10). Phenylpropane metabolism has important physiological significance in plants and is closely related to plant disease resistance [58,59,60]. Phenylpropane metabolites are chemical barriers that plants use to resist pathogenic microorganisms. The genes involved in the synthesis of phenylpropanes were upregulated in the experimental group, indicating that the accumulation of phenylpropanes in rice helps them adapt to the PS-MP-containing environment. Based on these results, phenylpropane metabolism and plant hormone signal transduction are the main metabolic pathways via which rice responds to MP exposure.

The downregulated genes were found to be enriched in linolenic acid metabolism and nitrogen metabolism pathways; the enrichment factors of these two pathways were the largest at 0.3 and 0.29, respectively (Figure 10). Four DEGs were involved in the biosynthesis of flavonoids and flavanols, six were involved in photosynthesis-antenna protein synthesis, and three were involved in sesquiterpene and triterpene biosynthesis pathways. The synthesis of these compounds may be related to the response of rice to PS-MP stress. Flavonoids are important compounds in most plants, with functions in plant growth, development, flowering, fruiting, antibacterial, and disease prevention [61]. Plants produce and accumulate large amounts of ROS when subjected to adversity stress, which destroys the normal structure and function of cells and may eventually lead to plant death [48]. Flavonoids are a major class of non-enzymatic active oxygen scavengers, among which flavanols have the strongest antioxidant properties [62]. Combined with the changes in antioxidant enzyme activity, several enzymatic active oxygen scavengers such as SOD and CAT, which are common in leaves, were significantly inhibited by high-concentration PS-MPs, and this led to a significant increase in the ROS content in roots. These results show that PS-MPs are stress factors to rice, and they can promote oxidative stress and induce other non-enzymatic antioxidants, further increasing the expression of genes involved in flavonoid biosynthesis.

In response to PS-MP pollution, rice specifically induced root flavonoid biosynthesis, enhanced the response and resistance to PS-MP pollution, and reduced the oxidative damage of plants caused by PS-MP pollution. Genes related to linolenic acid metabolism were downregulated; linolenic acid is one of the main fatty acids present in plant membrane lipids. In particular, the primary fatty acid in the membrane lipids of the plant photosynthetic membrane (thylakoid membrane) is linolenic acid (approximately 70%), the content of which is closely related to photosynthesis [63,64,65]. We found that the synthesis of plant cell membranes, especially thylakoid membranes, was inhibited by PS-MP treatment, which damaged the membrane structure. This indicates that PS-MPs may inhibit rice photosynthesis under stress.

## 4. Conclusions

The present work used the economically important rice crop as the experimental object to study the toxicity of MPs on rice seed germination. Our findings give further details on the impact of MPs on plants, particularly in the preliminary stage of development. The absorption and enrichment of MPs in rice seeds, buds, and roots were observed through electron microscopy. Indices including the germination rate and growth characteristic of rice seeds, antioxidant enzyme activity of buds, and ROS level of roots were measured, and transcriptional profiling in roots was performed. Based on the measured data, the toxic effects of MPs on rice germination and the response mechanism of rice to MPs were studied at both individual and molecular levels. The results show that PS-MPs had no significant effect on the germination of rice seeds (*p* > 0.05). However, PS-MPs significantly promoted root length (10 mg L^−1^; *p* < 0.05), and significantly reduced antioxidant enzyme activity (1000 mg L^−1^; *p* < 0.05), while inducing the rice root stress response. Through screening of transcriptome data, we also observed that MPs can upregulate genes related to plant root antioxidant activity, such as flavonoids and flavonol biosynthesis, as well as downregulate genes involved in linolenic acid and nitrogen metabolism pathways. Hence, MPs may impact the oxidant-antioxidant system of plant roots and induce stress responses. The results of the present study enhance our understanding of the absorption and toxicity responses to MPs in agricultural crops.

## Supplementary Materials

The following are available online at https://www.mdpi.com/article/10.3390/toxics9080179/s1, Figure S1: Correlation between samples, Figure S2: Characterization of microplastics via transmission electron microscopy (TEM), Figure S3: T-IR spectra of 200 nm polystyrene (PS) microbeads and their comparison with the standard spectra, Figure S4: Effects of different PS-MP concentrations on dry quality of rice seedlings, Figure S5: Scanning electron microscopy (SEM) analysis of microplastic enrichment on rice seed coat treated with 1000 mg L^−1^, Figure S6: Transmission electron microscopy (TEM) images of rice stem cells. Figure S7: Effects of different PS-MP concentrations at different times on the germination of rice seeds, Table S1: Zeta potential of microplastics in suspension, Table S2: Summary of sequence analysis.
