# Supplementary material for: Effect of Polystyrene Microplastics on Rice Seed Germination and Antioxidant Enzyme Activity
Source: Toxics. 2021 Jul 30;9(8):179. doi: 10.3390/toxics9080179 (PMC8402430; doi:10.3390/toxics9080179)
Supplement: Supplementary file 1 [file toxics-09-00179-s001.zip › toxics-1290654 - supplementary.pdf]

toxics

Supplementary Materials: Effect of Polystyrene Microplastics on Rice Seed Germination and Antioxidant Enzyme Activity

Qiuge Zhang, Mengsai Zhao, Fansong Meng, Yongli Xiao, Wei Dai and Yaning Luan

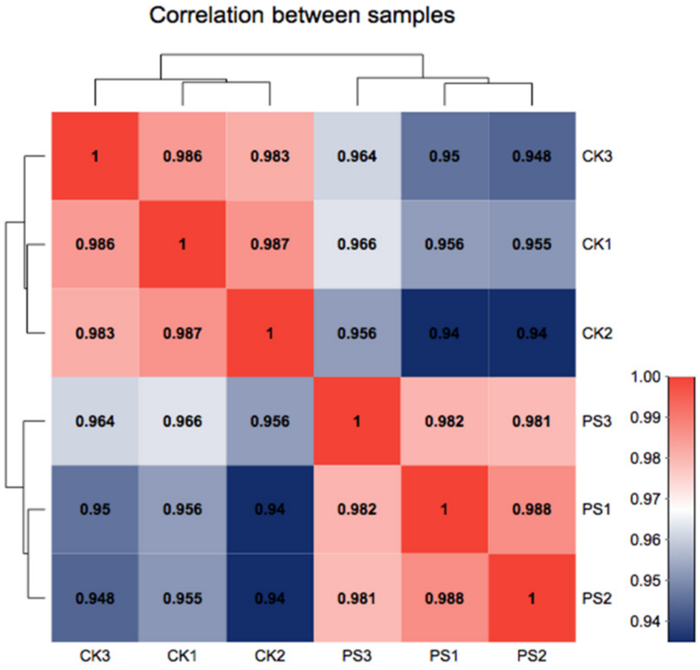

	CK3	CK1	CK2	PS3	PS1	PS2
CK3	1	0.986	0.983	0.964	0.95	0.948
CK1	0.986	1	0.987	0.966	0.956	0.955
CK2	0.983	0.987	1	0.956	0.94	0.94
PS3	0.964	0.966	0.956	1	0.982	0.981
PS1	0.95	0.956	0.94	0.982	1	0.988
PS2	0.948	0.955	0.94	0.981	0.988	1

Figure S1. Correlation between samples.

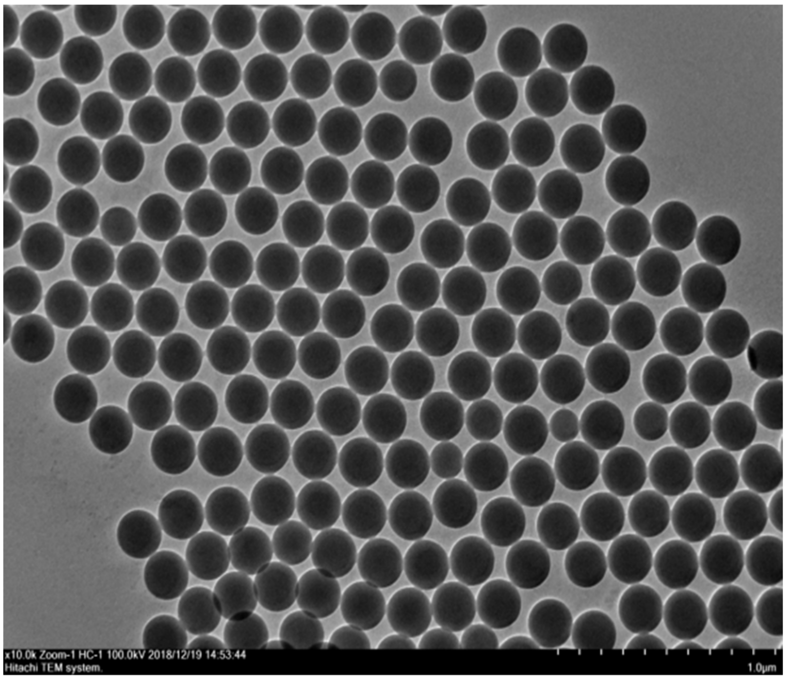

Figure S2. Characterization of microplastics via transmission electron microscopy (TEM).

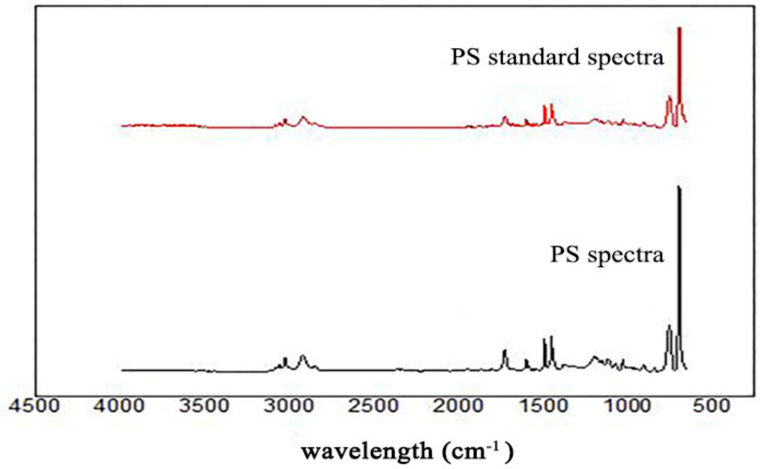

Figure S3. FT-IR spectra of 200 nm polystyrene (PS) microbeads and their comparison with the standard spectra.

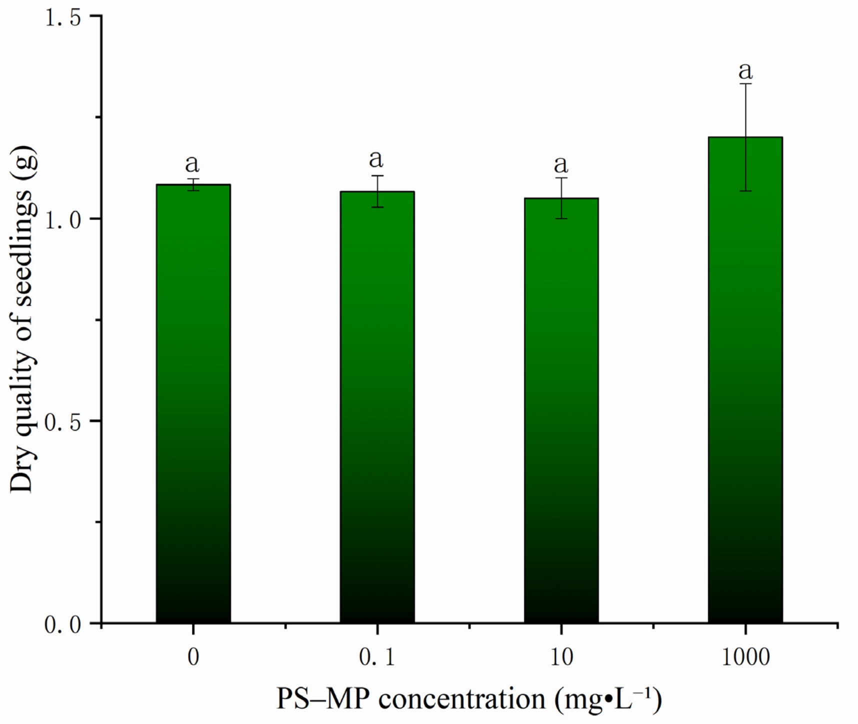

Figure S4. Effects of different PS-MP concentrations on dry quality of rice seedlings.

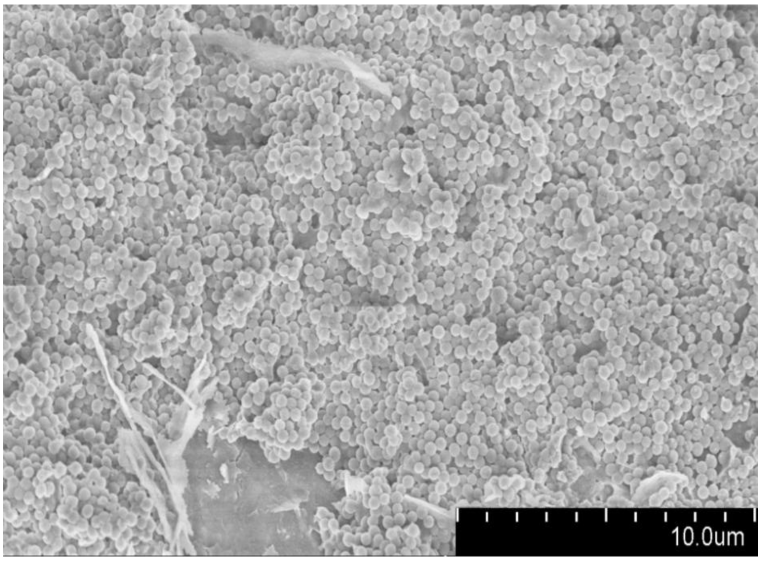

Figure S5. Scanning electron microscopy (SEM) analysis of microplastic enrichment on rice seed coat treated with 1000 mg L^{-1}.

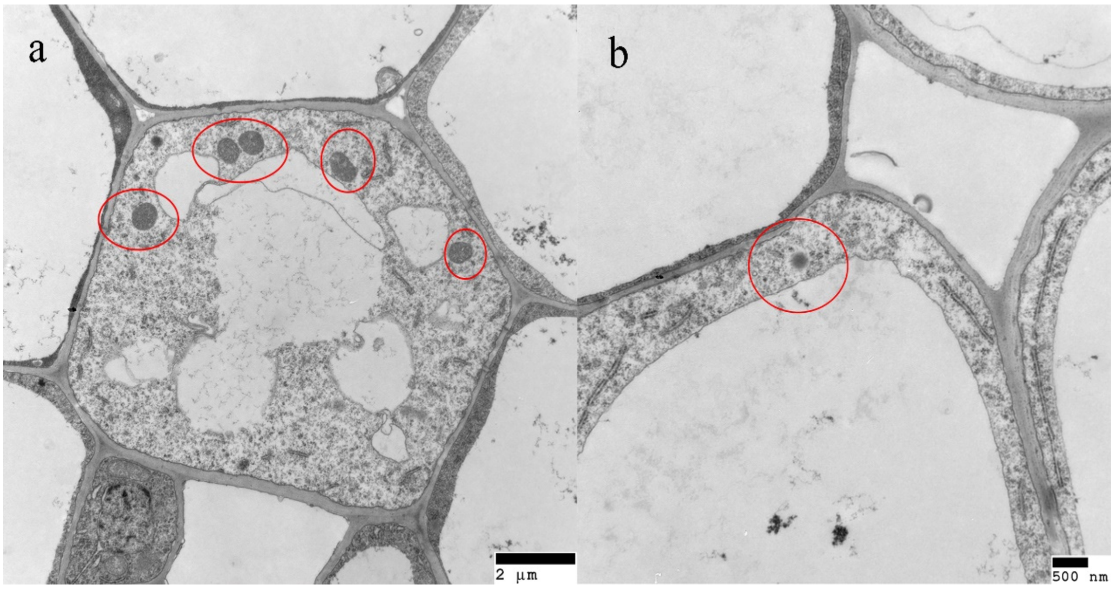

Figure S6. Transmission electron microscopy (TEM) images of rice stem cells. (**a**) control group; (**b**) 1000 mg L^{-1} treated group.

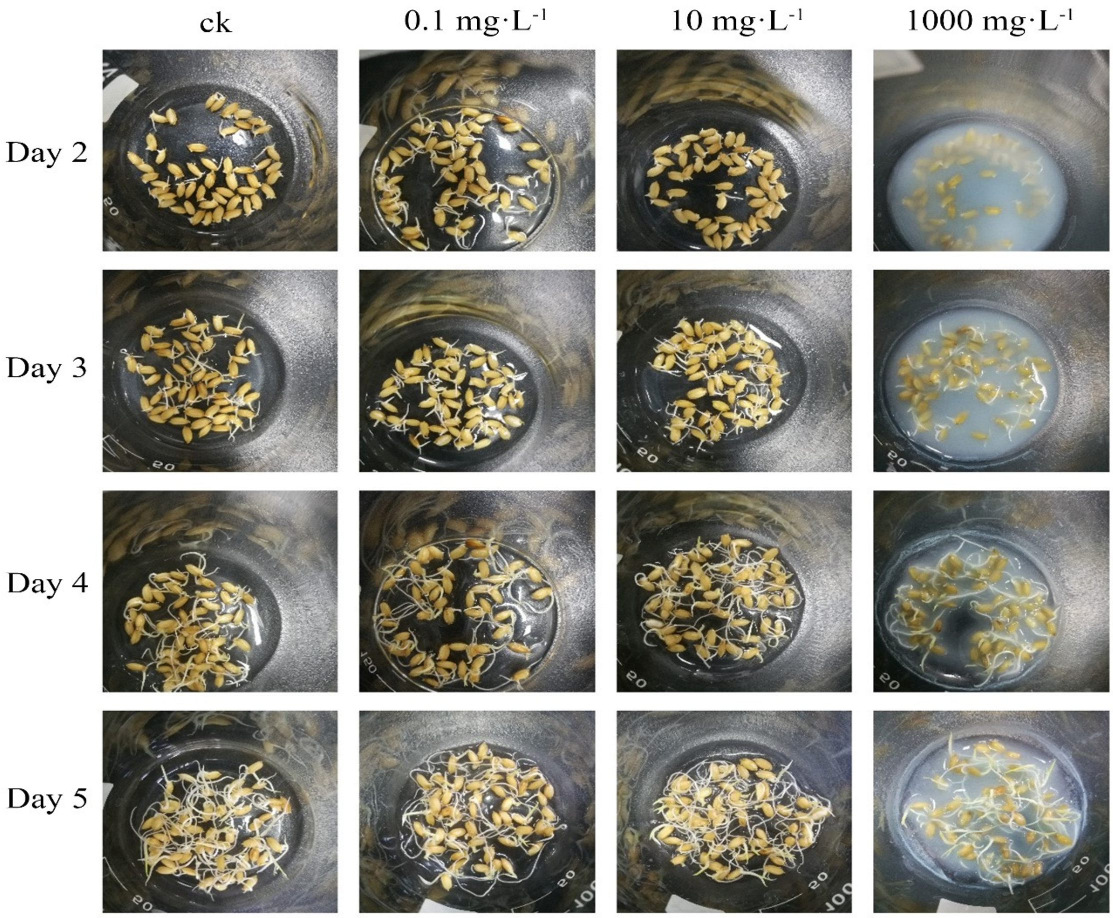

Figure S7. Effects of different PS-MP concentrations at different times on the germination of rice seeds. No germination is apparent on the first day, while that on the sixth and seventh days is consistent with the fifth day.

Table S1. Zeta potential of microplastics in suspension.

Nanoplastic Concentration, mg·L^{-1}	Zeta Potential, mV
0.1	−1.26
10	−37
1000	−48.2

Table S2. Summary of sequence analysis.

Sample	Raw Reads [a]	Clean Reads [b]	Clean Bases [c]	Error Rate (%) [d]	Q20(%) [e]	Q30(%) [e]	GC Content (%) [f]
CK1	48,235,008	47,679,212	7,147,291,640	0.0266	97.39	92.58	54.03
CK2	42,269,238	41,730,978	6,249,110,223	0.0269	97.27	92.32	54.12
CK3	41,997,918	41,516,362	6,216,283,751	0.0263	97.53	92.9	54.06
PS1	44,697,730	44,105,636	6,598,683,936	0.0269	97.27	92.35	53.41
PS2	44,650,666	44,139,948	6,603,119,692	0.0264	97.47	92.78	53.68
PS3	47,244,116	46,671,662	6,978,638,696	0.0265	97.44	92.72	53.6

[a] Counts for raw sequence data; [b] the calculation method is the same as Raw Reads, except that the statistical file is the filtered sequencing data; [c] the number of clean reads multiplied by the length and converted to G as the unit; [d] sequencing error rate (clean data); [e] Percentage of bases with Phred values greater than 20 and 30 compared to total bases (clean data); [f] The percentage of the total G and C based compared to the total number of bases (clean data).
